# Analysis of genomic copy number variations through whole-genome scan in Yunling cattle

**DOI:** 10.3389/fvets.2024.1413504

**Published:** 2024-07-22

**Authors:** Dong Dang, Lilian Zhang, Lutao Gao, Lin Peng, Jian Chen, Linnan Yang

**Affiliations:** ^1^College of Big Data, Yunnan Agricultural University, Kunming, China; ^2^Yunnan Engineering Technology Research Center of Agricultural Big Data, Kunming, China; ^3^Yunnan Engineering Research Center for Big Data Intelligent Information Processing of Green Agricultural Products, Kunming, China

**Keywords:** Yunling cattle, copy number variation, single-molecule sequencing, Vst, genome selection

## Abstract

Yunling cattle is a new breed of beef cattle bred in Yunnan Province, China, which has the advantages of fast growth, excellent meat quality, improved tolerance ability, and important landscape value. Copy number variation (CNV) is a significant source of gene structural variation and plays a crucial role in evolution and phenotypic diversity. Based on the latest reference genome ARS-UCD2.0, this study analyzed the genome-wide distribution of CNVs in Yunling cattle using short-read whole-genome sequencing data (*n* = 129) and single-molecule long-read sequencing data (*n* = 1), and a total of 16,507 CNVs were detected. After merging CNVs with overlapping genomic positions, 3,728 CNV regions (CNVRs) were obtained, accounting for 0.61% of the reference genome. The functional analysis indicated significant enrichment of CNVRs in 96 GO terms and 57 KEGG pathways, primarily related to cell adhesion, signal transduction, neuromodulation, and nutritional metabolism. Additionally, 111 CNVRs overlapped with 76 quantitative trait loci (QTLs), including Subcutaneous fat thickness QTL, Longissimus muscle area QTL, and Marbling score QTL. Several CNVR-overlapping genes, including *BZW1, AOX1*, and *LOC100138449*, overlap with regions associated with meat color and quality QTLs. Furthermore, Vst analysis showed that *PSMB4, ERICH1, SMC2*, and *PPP4R3A* were highly divergent between Yunling and Brahman cattle. In summary, we have constructed the genomic CNV map of Yunling cattle for the first time using whole-genome resequencing. This provides valuable genetic variation resources for the study of the Yunling cattle genome and contributes to the study of economic traits in Yunling cattle.

## 1 Introduction

The cattle (Bos taurus), since its domestication about 10,000 years ago, has been a multipurpose domestic animal, occupying an important position in the development of animal husbandry and holding significant importance in national economic growth. It provides human with livestock products such as milk, meat, and leather, and is also used for cultivation and transportation (1). Environmental factors, geographic isolation, and human activities have significantly impacted the genome of cattle, contributing to the development of modern cattle and determining their phenotype, adaptability, and productive performance. Genetic variants, such as single nucleotide polymorphisms (SNPs) and insertion deletions (INDELs), have been extensively studied in different cattle populations to understand cattle evolution, including population structure, selection, population history and gene introgression (2–4). Some candidate genes related to reproduction, meat, milk and environmental adaptation have been identified (5).

Copy number variation (CNV) is a significant aspect of genomic structural variation. It refers to duplications or deletions of genomic segments, ranging in size from 50 bp to several Mp, which vary among individuals or species (6). CNV can interfere with gene expression and exert a greater impact on phenotypes compared to SNPs (7, 8). The current methods for detecting CNVs include comparative genomic hybridization arrays (CGH arrays), SNP arrays (such as the Illumina BovineHD BeadChip and Illumina BovineSNP50 BeadChip), whole genone sequencing, and long-reads sequencing. While whole genone sequencing and long-reads sequencing offer higher precision breakpoints, sensitivity, and resolution compared to array technologies, limited studies have been conducted to detect CNVs in cattle genomes using long-reads sequencing due to its high cost (5).

The Yunling cattle is the fourth new breed of beef cattle with completely independent intellectual property rights bred by the Yunnan Academy of Grassland and Animal Science, and it is the first breed bred in China through the ternary crossbreeding method. Yunling cattle are a beef cattle breed that has been carefully cultivated for over 30 years. They are selected from three breeds of cattle: Brahman cattle, Murray Grey cattle, and Yunnan Yellow cattle, using crossbreeding selection. It is characterized by rapid growth, high reproductive survival rates, good heat tolerance, and excellent meat quality (9). To date, only a limited number of studies have investigated the genomic distinctions between Yunling cattle and other breeds of cattle at the level of SNPs and INDELs. These differences have identified candidate genes associated with growth, muscle development, neurotransmitter concentration, and heat tolerance (9, 10). However, the research on copy number variation in Yunling cattle only focuses on the impact of CNV of a single gene on growth traits, such as *VAMP7, DYNC1I2, PLA2G2A, SYT11*, etc. (11–14).

This study combines high-coverage short-read data and long-read data for genome-wide CNV analysis, with a view to generate a comprehensive CNV landscape of Yunling cattle, investigating and compare the diversity and population genetic characteristics of CNV regions (CNVRs) in Yunling cattle. In addition, we conducted in-depth analyses of CNV functions and explored the population genetic characteristics of CNV using selective sweep analysis. This laid the foundation for determining the formation mechanism of economically important traits in Yunling cattle and provided a theoretical basis for future Yunling cattle breeding.

## 2 Materials and methods

### 2.1 Samples collection and genome sequencing

In this study, we collected sequencing datasets of 130 Yunling cattle and 10 Brahman cattle (Supplementary Table 1). The short-sequencing datasets of 129 Yunnan Ling cattle and 10 Brahman cattle were downloaded from the NCBI public database under the BioProject accession number PRJNA555741. Another sample data was obtained from a 4-year-old male Yunling cattle reared at Chuxiong Jinda Farm, Chuxiong City, Yunnan Province. Genomic DNA was extracted from heart tissue using the standard phenol-chloroform extraction method for DNA sequencing library construction. The integrity of genomic DNA molecules was checked using agarose gel electrophoresis. The BGISEQ DNBSEQ-T7 platform was used for short sequencing (bp) to obtain 161.89 GB of raw data (64X coverage of the estimated genome size), and the PacBio Sequel II platform (CCS mode) was used for single-molecule long-read sequencing to obtain 61.81 GB of raw data for genome assembly. The sequencing work has performed at GrandOmics Biosciences Co., Ltd. (Wuhan, China).

### 2.2 Sequence alignment to reference genome

After obtaining the downloaded and sequenced raw data from the whole genome sequencing of Yunling cattle, fastp 0.23.4 (https://github.com/OpenGene/fastp) was used to filter the raw data for quality control and retain the relatively high quality sequencing data. The filtered data were then aligned to the latest cattle reference genome ARS-UCD2.0 downloaded from the Ensembl website using BWA-mem with default settings, and the PCR duplicates that could affect the CNV analysis were removed using Picard 2.9.2 (https://broadinstitute.github.io/picard/) and Markduplicates. Additionally, PacBio long-read were mapped to the cattle genome (ARS-UCD2.0) using minimap2 (15) with default settings.

### 2.3 Detection of CNVs and CNVRs

We used Lumpy and CNVcaller to identify CNVs in Illumina short-read sequencing data, and Sniffles (6) to identify CNVs in PacBio long-read sequencing data. We then merged the CNVcaller results with the Sniffles and lumpy results, aiming to maximize the retention of population-specific variants while reducing rare variants at the individual level. Finally, we obtained results based on CNVcaller corrected for individual PacBio data. Specific details are as follows: Sniffles (version: 1.0.10) was employed to detect structural variants (SVs) based on PacBio long reads with default parameters. The SV analysis outputs were filtered through three steps: (1) removed ambiguous breakpoints (flag: IMPRECISE) and low quality SVs; (2) removed SVs shorter than 50 bp; (3) SVs with less than four supporting reads were removed[8]. CNVs were detected using Lumpy software (v 0.2.13) with default parameters, and CNVs were generated by performing discordant-read pairs and split-read pairs on each sample through the lumpyexpress module. CNVcaller was then utilized to detect CNVs. Manual checking and SURVIVOR (version 0.0.1) (16) were used to combine the results of the three software to determine the final dataset. The CNVs of Yunling cattle is divided into duplication CNVR, deletion CVNR and duplication-deletion CNVR, and the length of CNVR is ≤ 50 kb (deletion and both), and the length of CNVR is <500 kb (duplication) (17). In addition, the distribution of these regions on the chromosomes of Yunling cattlewas analyzed using Bioconductor's RIdeogram software package (18).

### 2.4 Functional annotation and enrichment analysis of CNVRs

To elucidate the functions associated with the identified CNVs in the Yunling cattle genome, the Yunling cattle annotation file used in this study was downloaded from the NCBI database as ARS-UCD2.0, and the annotation of candidate CNVRs was performed using ANNOVAR (19). GO enrichment and Kyoto Encyclopedia of Genes and Genomes (KEGG) analyses were performed using the Database for Annotation, Visualization and Integrated Discovery (DAVID; https://david.ncifcrf.gov/) (20) for protein-coding genes only. Biological processes, cellular components and molecular functions were used as GO term categories with a significance level of *p*-value of 0.05. Additionally, cattle quantitative trait loci (QTL) were downloaded from the cattle QTLdb (https://www.animalgenome.org/cgi-bin/QTLdb/BT/summary) (21) and compared with the identified CNVRs. As there are no studies of relevant QTL by ARS-UCD2.0, we only considered QTL reported in ARS-UCD1.2 with confidence intervals <5 Mb. We used the Bedtools-v2.27.1 (22) “intersect” command to detect which QTL overlapped with identified CNVRs.

### 2.5 Sweep selective analysis of the CNVR

We calculated the Vst (23) between Yunling cattle and Brahman cattle to identify the highly differentiated regions between the two populations. Due to the large difference in the amount of data between the two populations, we randomly selected ten of the 130 Yunling cattle short-read data and one Yunling cattle long-read data to form the Yunling cattle dataset for Vst analysis. Vst is a method similar to the Fst method for calculating selection between populations and is therefore used to calculate data on population differences based on copy number. The formula is Vst = (Vt - Vs)/Vt, where Vt is the variance between all uncorrelated individuals and Vs is the average variance within each population, weighted according to population size (24). Subsequently, CNVRs with the top 10% of Vst values were then used as candidate CNVRs and functional enrichment analysis was performed on these regions.

## 3 Results

### 3.1 The landscape of copy number variation in Yunling cattle

**Figure 1:**
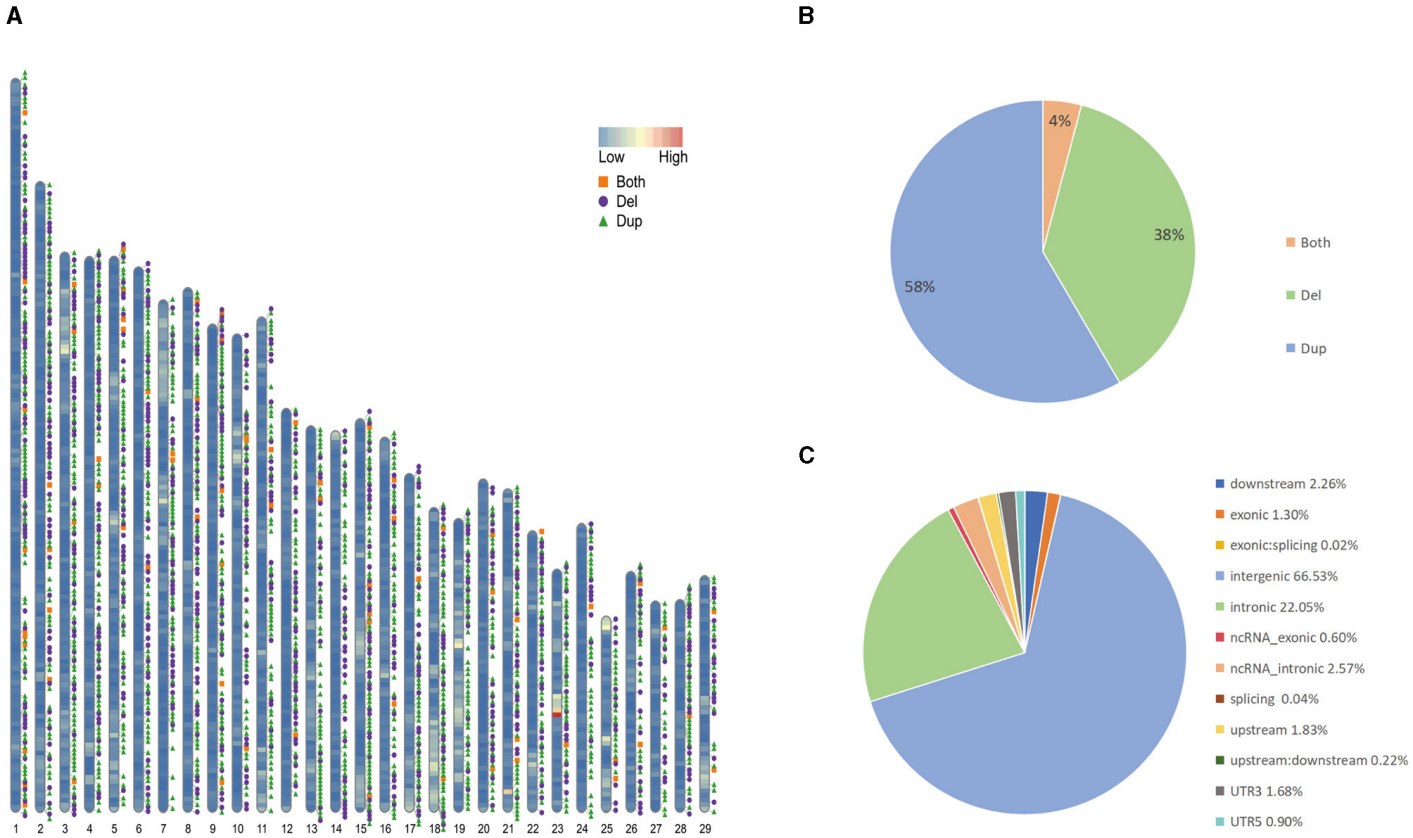
Genomic diversity and distribution of CNVR in Yunling cattle. **(A)** Autosomal distribution of CNVR. The colors painted on the chromosomes represent gene densities, and the different colored positions outside the chromosomes represent duplications (green), deletions (purple), and duplications and deletions (orange). **(B)** Frequency of different types of CNVRs. **(C)** Functional classification of detected CNVRs.

We collected sample data from 130 Yunling cattle and 10 Brahman cattle. Among the 140 samples, we newly performed long-read and short-read sequencing on 1 Yunling cattle sample and obtained 61.81 and 161.89 GB of raw data, respectively. The other 139 genome sequences are available online. Among them, the average coverage depths of 25 X and 64 X were obtained for the self-sequenced long and short data, and 4.2263 X to 7.529 X for the online-accessible data, and an average coverage of 99.73% was obtained after mapping the reads to the cattle reference genome ARS-UCD2.0 (Supplementary Table 1). An average coverage depth between 4x and 8x, as reported in the literature, can provide sufficient power for CNV detection using read depth-based methods (25). We constructed confidential CNV datasets by applying three software packages, Sniffles, lumpy and CNVcaller, to Nanopore long-read sequencing data and Illumina short-read sequencing data. We generated CNVR datasets for two cattle breeds, with a total of 16,507 CNVs detected in Yunling cattle. After merging CNVs with overlapping genomic locations, 3,728 CNV regions (CNVRs) were obtained (Figure 1A; Supplementary Table 2), including 2,175 duplication CNVRs, 1,401 deletion CNVRs, and 152 duplication and deletion CNVRs (Figure 1B), and a total of 3,633 CNVRs detected in Brahman cattle, including 2,186 duplication CNVRs; 1,321 deletion CNVRs; and 126 duplication and deletion CNVRs. Here, we focused on analyzing the CNVRs of Yunling cattle with a total length of 16,392,409 bp and an average length of 4,397 bp, covering 0.61% of the reference genome. The length of CNVRs was mainly distributed in the range of 1,000–2,000 bp, accounting for about 76% of the detected CNVRs (Supplementary Figure 1). Furthermore, the number of CNVRs decreased with increasing length (>500 bp). Furthermore, our results showed that CNVRs were unevenly distributed throughout the genome, with 66.5% of CNVRs (3,676) located in intergenic regions and only 1.3% in exonic regions (Figure 1C).

### 3.2 Functional annotation of CNVRs

**Figure 2:**
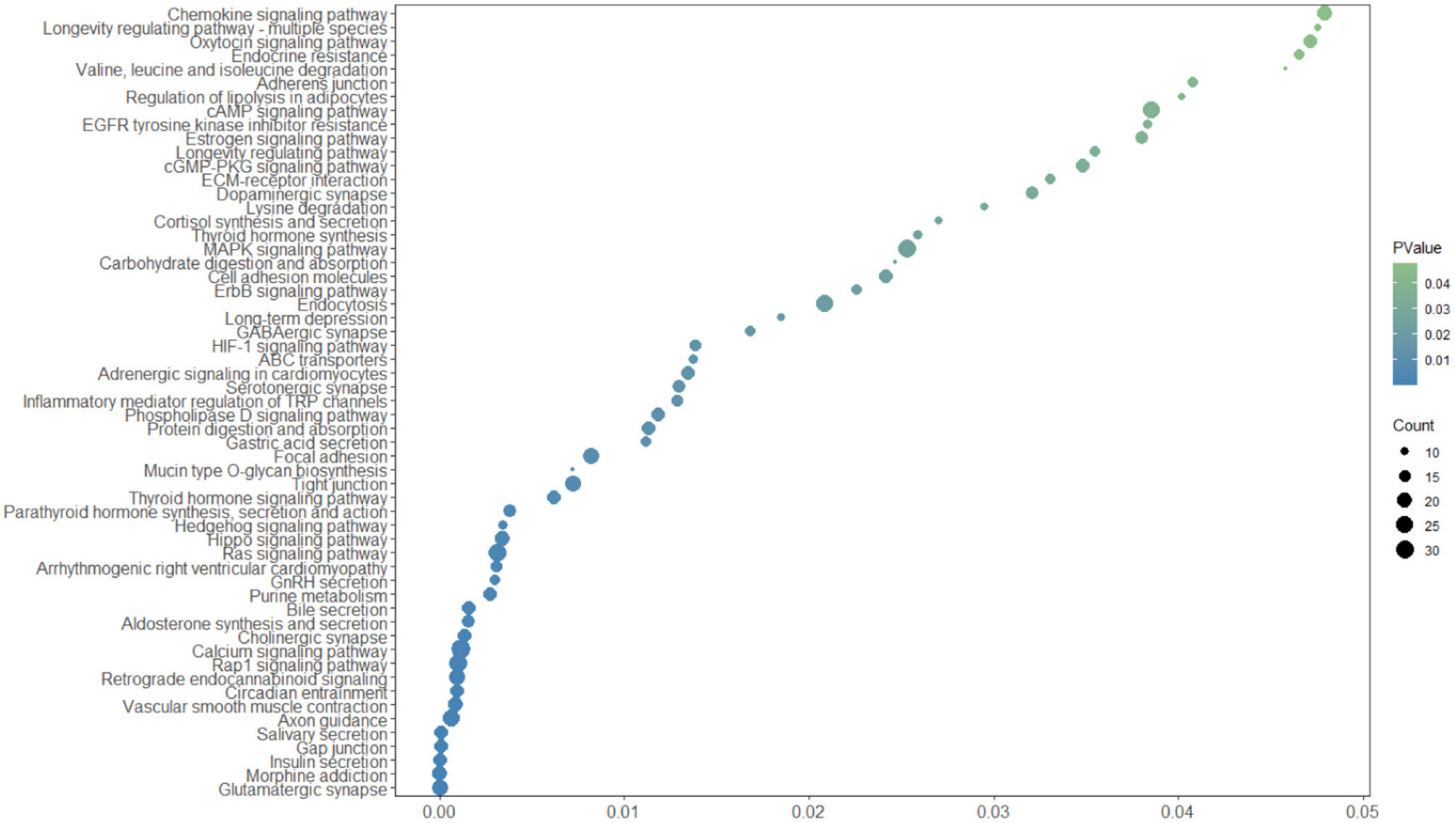
KEGG pathway enrichment analysis in Yunling cattle.

Genes annotated corresponding to 3,728 CNVRs using the NCBI ARS-UCD2.0 reference genome. When CNVRs and genes overlapped by more than 1 bp, the relevant gene was annotated, otherwise the closest gene was annotated. When other genes were present within 1kb, additional annotation was required because it could affect the expression of that gene. Eventually, 3,728 CNVRs were annotated to a total of 1,572 genes. In order to further understand the effects of CNV on various aspects of growth in Yunling cattle, this study used the DAVID website to perform GO enrichment analysis and KEGG pathway analysis on the genes in the CNVRs. The result showed 96 GO terms were enriched (*p*-value >0.01), including 35 biological processes, 36 cellular components and 25 molecular functions (Supplementary Table 3), which were involved in cell adhesion, neuromodulation, immunomodulation and metabolism. Specifically, ATP binding (GO:0005524), calcium ion binding (GO:0005509), signal transduction (GO:0007165), neuron projection (GO:0043005), actin binding (GO:0003779), etc. In addition, KEGG pathway analysis showed that CNVR-carrying genes were enriched in 57 pathways (*p*-value >0.05, Figure 2; Supplementary Table 4), including signal transduction (bta04014:Ras signaling pathway, bta04015:Rap1 signaling pathway, bta04022:cGMP- PKG signaling pathway, bta04024:cAMP signaling pathway and bta04020:Calcium signaling pathway), nutrient metabolism (bta00230:Purine metabolism, bta04911. Insulin secretion and bta04974:Protein digestion and absorption), Regulation of lipolysis in adipocytes (bta04923) and ABC transporters (bta02010).

### 3.3 QTLs overlapping with identified CNVRs

In order to further reveal the CNVRs associated with economic traits in Yunling cattle and to verify their genetic effects, the detected CNVRs were compared with QTLs in the cattle QTL database. The results showed that 111 CNVRs overlapped with 76 QTLs by more than 1 kb, including Subcutaneous fat thickness QTL (17 CNVRs), Longissimus muscle area QTL (16 CNVRs), Multiple birth QTL (12 CNVRs), Marbling score QTL (8 CNVR), meat color and quality QTL (10 CNVRs) (Supplementary Table 5). Some CNRV genes associated with economic performance of Yunling cattle were identified, such as *XKR4, ZBTB7C, CCDC15* genes relevant to Longissimus muscle area QTL. *NHLRC3, LOC132342211, SLCO3A1* genes relevant to Marbling score QTL. *BZW1, AOX1, LOC100138449* genes linked with meat quality QTL.

### 3.4 CNVRs diverging among populations

**Figure 3:**
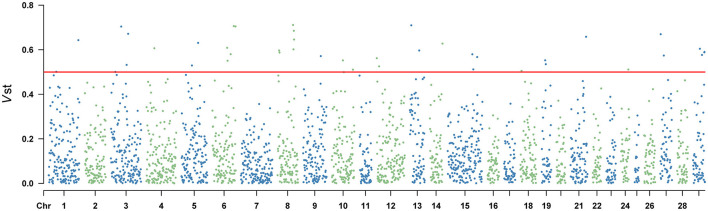
Vst values of the autosomal copy number variation region (CNVR) in Yunling cattle and Brahman cattle with a average Vst of 0.1268.

Vst statistic was utilized to analyze the population differentiation of CNVR between Yunling cattle and Brahman cattle. The method was similar to Fst in estimating population-specific selection pressure at the gene level. However, Vst showed the average value of detected response CNVR reached 0.3061 by utilizing CNVR-annotated protein-coding genes (Supplementary Table 6). As shown in Figure 3 and Supplementary Table 6, the different CNVRs were unevenly distributed across the chromosomes, and the Manhattan plot showed the results of Vst with the chromosomes in the horizontal coordinates and the VST values in the vertical coordinates. To understand the genes with a high degree of variation between varieties, genes with Vst >0.5 (up to 98th percentile) were examined. Result revealed that there were 10 CNVR overlapping genes or loci, including *LOC101906606, LOC132346850, LOC112447126, POLN, LOC615258, SMC2, PSMB4, ERICH1, PPP4R3A, LARGE1*. Remarkably, *PSMB4, ERICH1, SMC2*, and *PPP4R3A* genes play crucial roles in growth and development.

## 4 Discussion

During domestication and diversification, the frequency of CNV in the genomes of species responds to selection pressures. Considerable effort has been devoted to identifying causal mutations and genes. However, screening selected genomic copy number genetic markers is complex. Although population genetics in cattle has been extensively studied based on SNPs, little is known about the effects of CNVs on phenotypic and evolutionary traits. CNVs cover a larger region of the genome than SNPs and can affect gene function in a number of ways, including altering gene structure and dosage, altering gene regulation and exposing recessive alleles (26). Over the past decades, high-throughput sequencing technologies and bioinformatics tools have been increasingly used to construct genome-wide CNV profiles (17). CNV diversity has been extensively explored in Bos Taurus, Bos Indicus, and their crossing populations (27).

Yunling cattle have good fattening performance, significant body proportions, high meat yield, good carcass traits and good fatty acid composition in meat, which is an important source of beef production in China (28). In our study, we firstly used different sequencing platforms (short-reads and long-reads) to sequence high quality whole genome data of 140 Yunling and Brahman cattle to detect CNV with a high pairwise ratio (average pairwise ratio: 99.73%) compared to the newly reported reference genome (ARS-UCD2.0) (Supplementary Table 1). compared to the UMD 3.1 reference genome. Its improved reliability in screening CNVs (29). Short-reads sequencing has high base identification accuracy, which is an advantage in detecting shorter structural variants, but has an inherent disadvantage of exhibiting a high false-positive rate when detecting complex or long CNVs (30). Long-reads sequencing has the potential to substantially improve the reliability and resolution of structural variant detection. With an average read length of 10 kbp or more, reads can be more confidently compared to repetitive sequences that often mediate the formation of structural variants. Long-reads sequencing are also more likely to cross structural variant breakpoints with high confidence comparisons. However, long-reads sequencing also have the disadvantage of a high error rate in sequencing (31). Therefore, it happens that long and short reads data complement each other perfectly, providing us with the accuracy of detecting CNVs. We used three software programs for CNV detection, which used different algorithms to obtain accurate estimates of copy number at breakpoints and structurally variable sites, to construct a confidential CNV dataset for Yunling cattle, which also ensured that we obtained a highly confidential CNV dataset. In total, we detected 3,728 CNVRs in 130 Yunling cattle (Figure 1A) and 3,633 CNVRs in 10 Brahman cattle, which are similar to the CNVR levels in other cattle breeds (32). For better statistics, the variants were classified into three categories: duplication, deletion and duplication-deletion. The number of duplication was higher than the number of deletions (Supplementary Table 2). And the length of most CNVRs ranged from 1 to 2 kb (Supplementary Figure 1). In addition, the locations of CNVRs were not uniformly distributed in the cattle genome and they were not randomly distributed on chromosomes. Annotation revealed that CNVRs were mostly annotated in intergenic or intronic regions of the cattle genome (Figure 1C). Previous studies also support that many CNVRs are located on highly variable genes (17).

In this study, the ANNOVAR tool was used to identify genes located within CNVRs, and then DAVID was used to search for GO terms and KEGG pathway information for genes contained within CNVRs, and the purpose of obtaining these results was to speculate on the functions of these genes and the possible effects of CNVRs on economic traits in Yunling cattle. Existing studies have found that genes in CNVRs are mainly involved in immunity, sensory perception of the external environment (involving olfaction, vision, and taste), response to stimuli, and neurodevelopment, with relatively little involvement with nucleic acid binding, metabolism, and cell proliferation (33). This may be due to the fact that the emergence of genes with important functions CNV is the result of strong purifying selection. When CNVs occur in coding regions where the affected gene plays an important role in growth, it is highly likely that the occurrence of the variant will be harmful to the organism and therefore quickly eliminated. This is consistent with the results of our analyses. Three thousand seven hundred and twenty-eight CNVRs were annotated to a total of 1,572 genes. GO analyses revealed that these genes were mainly cell adhesion, neuromodulation, immunomodulation, and metabolism, while a small number of genes were involved in cell differentiation and organ development (Supplementary Table 3). Independent CNVs between different breeds may contribute to breed differences to varietal differences (34). In KEGG pathway analysis, CNVR genes were significantly enriched in signal transduction, nutrient metabolism, and ABC transporter proteins (Figure 2; Supplementary Table 4). Studies in mammals have revealed that ABC transporter proteins can carry a variety of endogenous metabolites, such as amino acids, peptides, and sugars, across lipid membranes, thereby facilitating the absorption and utilization of these nutrients (35).

The CNVRs detected in this study were compared with the QTLs reported in the cattle QTL database. It was found that QTL affecting economic traits of livestock genetic variation can be identified by screening the genome for relevant genes contained in the CNVR (36). After integrating CNVR into QTL, we identified 111 CNVR overlapping with 76 cattle QTL regions in this study (Supplementary Table 5). Many of the CNV overlapping genes, such as *XKR4, ZBTB7C, CCDC15, AOX1*, were located in growth and carcass QTL regions.The *XKR4* gene was identified to be associated with growth traits especially Heritabilities for carcass weight (37, 38). The *ZBTB7C* gene controlled the expression of *MMPS*, which is the zinc-containing endopeptidases that play roles in cell proliferation, migration, differentiation, angiogenesis, and apoptosis (39). *CCDC15*'s role in recruiting both the inner scaffold protein *POC1B* and the distal *SFI1*/Centrin-2 complex to centrioles (40). The levels of 2-pyrrolidone and glycerophospholipids are regulated by the gene expression of *AOX1*, which further affects the levels of volatiles, 2-pyrrolidone and decanal, respectively (41). *NHLRC3, LOC132342211*, and *SLCO3A1*, are located in the marbling scoring QTL region. Therefore, these identified CNV-carrying genes provide candidate molecularly relevant markers for future Yunling cattle breeding.

Selective scanning can reveal putative regions subject to environmental and artificial selection during local adaptation and domestication (9). In genomes, calculation of paired Vst values can be used to screen for key CNVRs that differ significantly between populations (42). In this study, five CNVRs carrying the *PSMB4, POLN, LARGE1, SMC2* and *PPP4R3A* genes showed significant pairwise differentiation between Yunling Cattle and Brahman cattle (Figure 3; Supplementary Table 6). *PSMB4* has been reported as a key gene regulating muscle growth and development, which determines postnatal growth rate, muscle fiber diameter and density, and fiber type in pigs (43). This function of *PSMB4* gene may play an important role in good carcass traits in Yunling cattle. Subsequently, Yang et al. also demonstrated that *PSMB4* overexpression inhibited cardiomyocyte apoptosis and *I* κ*B*α expression, promoted the activation of *NF-*κ*B* (44). DNA polymerase v (pol v), encoded by the *PLON* gene, is an A-family DNA polymerase in vertebrates and some other animal lineages. Takata et al. showed that a function of pol v in meiotic homologous recombination in processing specific substratess (45). A previous study found that Large myd/Largemyd (myd) mice lack expression of like-acetylglucosaminyltransferase-1 (*Large1*) and exhibit severe muscle pathophysiology, impaired mobility, and a markedly reduced life span (46). The *SMC2* gene is considered a candidate gene associated with growth and meat production traits in sheep (47). The Protein phosphatase 4 regulatory subunit 3A (*PPP4R3A*), forms a highly conserved trimeric complex called protein phosphatase 4 (*PP4*) with *PPP4C* and *PPP4R2*. This complex plays a role in regulating the cellular processes, including DNA damage repair. *PPP4R3A* is widely expressed in various tissues and organs, participating in multiple cellular functions, such as cell proliferation, apoptosis, and cell cycle regulation (48). Some of the genes associated with physical traits in Yunling cattle were artificially selected in a targeted manner during domestication. Thus, under these selective pressures, CNVs may accumulate in Yunling cattle populations, thus forming the genetic basis for economically important traits.

## 5 Conclusion

In current study, we conducted comprehensive analyses to explore genetic variation in Yunling cattle. Based on a high-quality cattle reference genome, we constructed a CNV map of Chinese Yunling cattle using whole genome resequencing data. We defined common and breed-specific CNVRs and further analyzed the possible functions of overlapping CNVR genes using enrichment analysis and QTL database search. Based on paired Vst statistics, we examined CNVR-based population differentiation between Yunling cattle and Brahman cattle and revealed potential genomic regions that may be subject to selection. Our results provide a valuable resource for genome-wide variation in Yunling cattle and help to elucidate the genetic basis of superior traits in Yunling cattle. In addition, these results will contribute greatly to the future selection and development of economic traits in Yunling cattle.

## Data availability statement

The datasets presented in this study can be found in online repositories. The names of the repository/repositories and accession number(s) can be found in the article/Supplementary material.

## Ethics statement

The animal study was approved by Yunnan Agricultural University Life Sciences Ethics Committee. The study was conducted in accordance with the local legislation and institutional requirements.

## Author contributions

DD: Writing – original draft. LZ: Data curation, Writing – review & editing. LG: Supervision, Writing – review & editing. LP: Methodology, Writing – review & editing. JC: Validation, Writing – review & editing. LY: Funding acquisition, Resources, Writing – review & editing.

## References

[1] Magee DA MacHugh DE Edwards CJ. Interrogation of modern and ancient genomes reveals the complex domestic history of cattle. Anim. Front. (2014) 4:7–22. 10.2527/af.2014-001732704858

[2] Decker JE McKay SD Rolf MM Kim J Molina Alcalá A Sonstegard TS. Worldwide patterns of ancestry, divergence, and admixture in domesticated cattle. PLoS Genet. (2014) 10:e1004254. 10.1371/journal.pgen.1004254 24675901 PMC3967955

[3] Kim J Hanotte O Mwai OA Dessie T Bashir S Diallo B. The genome landscape of indigenous African cattle. Genome Biol. (2017) 18:1–14. 10.1186/s13059-017-1153-y 28219390 PMC5319050

[4] Kim K Kwon T Dessie T Yoo D Mwai OA Jang J. The mosaic genome of indigenous African cattle as a unique genetic resource for African pastoralism. Nat Genet. (2020) 52:1099–110. 10.1038/s41588-020-0694-2 32989325

[5] Sun T Pei S Liu Y Hanif Q Xu H Chen N. Whole genome sequencing of simmental cattle for SNP and CNV discovery. BMC Genom. (2023) 24:179. 10.1186/s12864-023-09248-x 37020271 PMC10077681

[6] Mills RE Walter K Stewart C Handsaker RE Chen K Alkan C. Mapping copy number variation by population-scale genome sequencing. Nature. (2011) 470:59–65. 10.1038/nature09708 21293372 PMC3077050

[7] Weischenfeldt J Symmons O Spitz F Korbel JO. Phenotypic impact of genomic structural variation: insights from and for human disease. Nat Rev Genet. (2013) 14:125–38. 10.1038/nrg3373 23329113

[8] Xu Y Shi T Cai H Zhou Y Lan X Zhang C. Associations of MYH3 gene copy number variations with transcriptional expression and growth traits in Chinese cattle. Gene. (2014) 535:106–11. 10.1016/j.gene.2013.11.057 24316128

[9] Chen Q Zhan J Shen J Qu K Hanif Q Liu J. Whole-genome resequencing reveals diversity, global and local ancestry proportions in yunling cattle. J Anim Breed Genet. (2020) 137:641–50. 10.1111/jbg.12479 32297417

[10] Jia P Cai C Qu K Chen N Jia Y Hanif Q. Four novel SNPs of MYO1A gene associated with heat-tolerance in Chinese cattle. Animals. (2019) 9:964. 10.3390/ani9110964 31766183 PMC6912737

[11] Liu X Yang P Sun H Zhang Z Cai C Xu J. CNV analysis of VAMP7 gene reveals variation associated with growth traits in Chinese cattle. Anim Biotechnol. (2023) 34:1095–101. 10.1080/10495398.2021.2011741 35236249

[12] Li X Ding X Liu L Yang P Yao Z Lei C. Copy number variation of bovine DYNC1I2 gene is associated with body conformation traits in Chinese beef cattle. Gene. (2022) 810:146060. 10.1016/j.gene.2021.146060 34740731

[13] Yang H Yue B Yang Y Tang J Yang S Qi A. Distribution of copy number variation in syt11 gene and its association with growth conformation traits in Chinese cattle. Biology. (2022) 11:223. 10.3390/biology11020223 35205089 PMC8869484

[14] Yang P Cai C Niu M Liu X Wang H Liang H. Effect of copy number variation of PLA2G2A gene to growth traits in Chinese cattle. Gene. (2022) 809:146014. 10.1016/j.gene.2021.146014 34655722

[15] Li H. Minimap2: pairwise alignment for nucleotide sequences. Bioinformatics. (2018) 34:3094–100. 10.1093/bioinformatics/bty191 29750242 PMC6137996

[16] Jeffares DC Jolly C Hoti M Speed D Rallis C Dessimoz C. Transient structural variations alter gene expression and quantitative traits in Schizosaccharomyces pombe. bioRxiv [preprint]. (2016) 047266. 10.1101/047266

[17] Huang Y Li Y Wang X Yu J Cai Y Zheng Z. An atlas of CNV maps in cattle, goat and sheep. Sci China Life Sci. (2021) 64:1747–64. 10.1007/s11427-020-1850-x 33486588

[18] Hao Z Lv D Ge Y Shi J Weijers D Yu G. RIdeogram: drawing SVG graphics to visualize and map genome-wide data on the idiograms. PeerJ Comp Sci. (2020) 6:e251. 10.7717/peerj-cs.251 33816903 PMC7924719

[19] Wang K Li M Hakonarson H. ANNOVAR functional annotation of genetic variants from high-throughput sequencing data. Nucleic Acids Res. (2010) 38:e164. 10.1093/nar/gkq603 20601685 PMC2938201

[20] Huang DW Sherman BT Lempicki RA. Systematic and integrative analysis of large gene lists using DAVID bioinformatics resources. Nat Protoc. (2009) 4:44–57. 10.1038/nprot.2008.211 19131956

[21] Hu ZL Park CA Reecy JM. Building a livestock genetic and genomic information knowledgebase through integrative developments of Animal QTLdb and CorrDB. Nucleic Acids Res. (2019) 47:D701–10. 10.1093/nar/gky1084 30407520 PMC6323967

[22] Quinlan AR Hall IM. BEDTools: a flexible suite of utilities for comparing genomic features. Bioinformatics. (2010) 26:841–2. 10.1093/bioinformatics/btq033 20110278 PMC2832824

[23] Redon R Ishikawa S Fitch KR Feuk L Perry GH Andrews TD. Global variation in copy number in the human genome. Nature. (2006) 444:444–54. 10.1038/nature05329 17122850 PMC2669898

[24] Yang L Xu L Zhu B Niu H Zhang W Miao J. Genome-wide analysis reveals differential selection involved with copy number variation in diverse Chinese cattle. Sci Rep. (2017) 7:14299. 10.1038/s41598-017-14768-0 29085051 PMC5662686

[25] Bickhart DM Hou Y Schroeder SG Alkan C Cardone MF Matukumalli LK. Copy number variation of individual cattle genomes using next-generation sequencing. Genome Res. (2012) 22:778–90. 10.1101/gr.133967.111 22300768 PMC3317159

[26] Zhang F Gu W Hurles ME Lupski JR. Copy number variation in human health, disease, and evolution. Annu Rev Genom Hum Genet. (2009) 10:451–81. 10.1146/annurev.genom.9.081307.164217 19715442 PMC4472309

[27] Liu Y Mu Y Wang W Ahmed Z Wei X Lei C. Analysis of genomic copy number variations through whole-genome scan in Chinese Qaidam cattle. Front Vet Sci. (2023) 10:1148070. 10.3389/fvets.2023.1148070 37065216 PMC10103646

[28] Chen J Zhang L Gao L Wei Z Dang D Yang L. Population structure and genetic diversity of Yunling cattle determined by whole-genome resequencing. Genes. (2023) 14:2141. 10.3390/genes14122141 38136963 PMC10742670

[29] Zhou J Liu L Reynolds E Garrick D Shi Y. Discovering copy number variation in dual-purpose Xinjiang brown cattle. Front Genet. (2022) 12:747431. 10.3389/fgene.2021.747431 35222511 PMC8873982

[30] Qin P Lu H Du H Wang H Chen W Chen Z. Pan-genome analysis of 33 genetically diverse rice accessions reveals hidden genomic variations. Cell. (2021) 184:3542–58. 10.1016/j.cell.2021.04.046 34051138

[31] Sedlazeck FJ Rescheneder P Smolka M Fang H Nattestad M Von Haeseler A. Accurate detection of complex structural variations using single-molecule sequencing. Nat Methods. (2018) 15:461–8. 10.1038/s41592-018-0001-7 29713083 PMC5990442

[32] Mei C Junjvlieke Z Raza SHA Wang H Cheng G Zhao C. Copy number variation detection in Chinese indigenous cattle by whole genome sequencing. Genomics. (2020) 112:831–6. 10.1016/j.ygeno.2019.05.023 31145994

[33] De Smith A Walters R Froguel P Blakemore A. Human genes involved in copy number variation: mechanisms of origin, functional effects and implications for disease. Cytogenet Genome Res. (2009) 123:17–26. 10.1159/000184688 19287135 PMC2920180

[34] Paudel Y Madsen O Megens HJ Frantz LA Bosse M Bastiaansen JW. Evolutionary dynamics of copy number variation in pig genomes in the context of adaptation and domestication. BMC Genom. (2013) 14:1–13. 10.1186/1471-2164-14-449 23829399 PMC3716681

[35] Shi H Li T Su M Wang H Li Q Lang X. Identification of copy number variation in Tibetan sheep using whole genome resequencing reveals evidence of genomic selection. BMC Genom. (2023) 24:555. 10.1186/s12864-023-09672-z 37726692 PMC10510117

[36] Hu ZL Park CA Reecy JM. Bringing the animal QTLdb and CorrDB into the future: meeting new challenges and providing updated services. Nucleic Acids Res. (2022) 50:D956–61. 10.1093/nar/gkab1116 34850103 PMC8728226

[37] Alam MZ Haque MA Iqbal A Lee YM Ha JJ Jin S. Genome-wide association study to identify QTL for carcass traits in Korean Hanwoo Cattle. Animals. (2023) 13:2737. 10.3390/ani13172737 37685003 PMC10486602

[38] Bejarano DH Martínez RA Rocha JF. Genome-wide association study for growth traits in Blanco Orejinegro and Romosinuano cattle. Trop Anim Health Prod. (2023) 55:358. 10.1007/s11250-023-03743-9 37848724 PMC10581918

[39] Jeon BN Yoon JH Kim MK Choi WI Koh DI Hur B. Zbtb7c is a molecular ‘off' and ‘on' switch of Mmp gene transcription. Biochim Biophys Acta. (2016) 1859:1429–39. 10.1016/j.bbagrm.2016.09.004 27646874

[40] Arslanhan MD Cengiz-Emek S Odabasi E Steib E Hamel V Guichard P. CCDC15 localizes to the centriole inner scaffold and controls centriole length and integrity. J Cell Biol. (2023) 222:e202305009. 10.1083/jcb.202305009 37934472 PMC10630097

[41] Liu D Zhang H Yang Y Liu T Guo Z Fan W. Metabolome-based genome-wide association study of duck meat leads to novel genetic and biochemical insights. Adv Sci. (2023) 10:2300148. 10.1002/advs.202300148 37013465 PMC10288243

[42] Yuan C Lu Z Guo T Yue Y Wang X Wang T. A global analysis of CNVs in Chinese indigenous fine-wool sheep populations using whole-genome resequencing. BMC Genom. (2021) 22:1–10. 10.1186/s12864-021-07387-7 33485316 PMC7825165

[43] Li Q Hao M Zhu J Yi L Cheng W Xie Y. Comparison of differentially expressed genes in longissimus dorsi muscle of Diannan small ears, Wujin and landrace pigs using RNA-seq. Front Vet Sci. (2023) 10:1296208. 10.3389/fvets.2023.1296208 38249550 PMC10796741

[44] Yang C Yu P Yang F He Q Jiang B Zheng L. PSMB4 inhibits cardiomyocyte apoptosis via activating NF-κB signaling pathway during myocardial ischemia/reperfusion injury. J Mol Histol. (2021) 52:693–703. 10.1007/s10735-021-09977-x 33954843

[45] Takata Ki Reh S Yousefzadeh MJ Zelazowski MJ Bhetawal S Trono D. Analysis of DNA polymerase ν function in meiotic recombination, immunoglobulin class-switching, and DNA damage tolerance. PLoS Genet. (2017) 13:e1006818. 10.1371/journal.pgen.1006818 28570559 PMC5472330

[46] Yonekawa T Rauckhorst AJ El-Hattab S Cuellar MA Venzke D Anderson ME. Large1 gene transfer in older myd mice with severe muscular dystrophy restores muscle function and greatly improves survival. Sci Adv. (2022) 8:eabn0379. 10.1126/sciadv.abn0379 35613260 PMC9132445

[47] Zhang L Liu J Zhao F Ren H Xu L Lu J. Genome-wide association studies for growth and meat production traits in sheep. PLoS ONE. (2013) 8:e66569. 10.1371/journal.pone.0066569 23825544 PMC3692449

[48] Hu Y Han Z Guo H Zhang N Shen N Jiang Y. Identification of a novel germline PPP4R3A missense mutation Asp409Asn on familial non-medullary thyroid carcinoma. Biomedicines. (2024) 12:244. 10.3390/biomedicines12010244 38275415 PMC10813271

