# Supplementary figures and images for: Analysis of genomic copy number variations through whole-genome scan in Yunling cattle
Source: Front Vet Sci. 2024 Jul 22;11:1413504. doi: 10.3389/fvets.2024.1413504 (PMC11298805; doi:10.3389/fvets.2024.1413504)

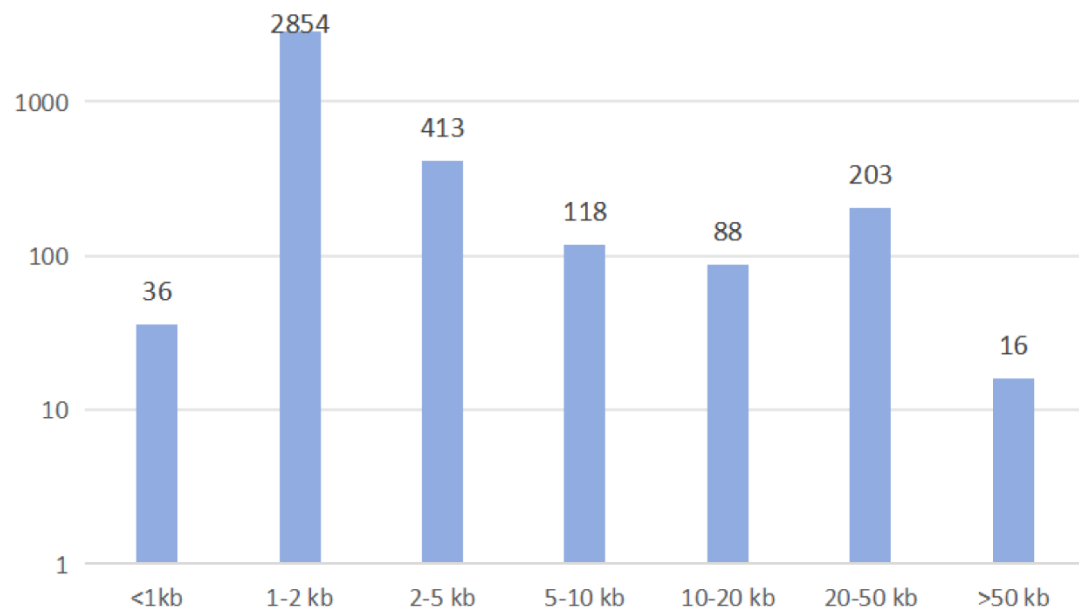

**Supplementary Figure 1 CNVR length distribution**

Supplement: Supplementary file 7 [file Image_1.pdf]
